# Spraying Effects of UAV Application on Droplet Effectiveness in Two Vine Trellis Systems of High-Slope Terrace Vineyards

**DOI:** 10.3390/plants14101452

**Published:** 2025-05-13

**Authors:** Zhao Le, Gastón Gutiérrez-Gamboa, Mengde Dong, Wei Zheng, Baoshan Sun

**Affiliations:** 1 School of Functional Food and Wine, Shenyang Pharmaceutical University, Shenyang 110016, China; myopt23@163.com (Z.L.); dongmengde0422@163.com (M.D.); 2 Instituto de Investigaciones Agropecuarias, INIA Carillanca, km 10 Camino Cajón-Vilcún s/n, Temuco Casilla Postal 929, Chile; gaston.gutierrez@inia.cl; 3 Escuela de Agronomía, Facultad de Ciencias, Ingeniería y Tecnología, Universidad Mayor, Temuco P.O. Box 54-D, Chile; 4 Pólo Dois Portos, Instituto Nacional de Investigação Agrária e Veterinária, I.P., Quinta da Almoinha, 2565-191 Dois Portos, Portugal

**Keywords:** drone, VSP trellis system, Y-shaped trellis system, vineyard spray, foliar application, manual spray

## Abstract

The application of unmanned aerial vehicles (UAVs) in viticulture is becoming increasingly popular. To the best of our knowledge, there are no studies comparing the effects of UAV spraying to manual spraying in high-slope vineyards. The goal of this study was to evaluate the effects of UAV spraying on droplet average diameter, droplet area percentage, and droplet density for vines grown using vertical shoot positioning (VSP) and Y-shaped trellis systems and to compare them with the effects of manual spraying via an electric knapsack sprayer. The results showed that manual spraying led to the greatest area of droplets for the VSP trellis system, and the uniformity and penetration of droplets with the UAV spraying method were higher than those for manual spraying for this trellis system. Regarding the Y-shaped trellis system, the UAV spraying method yielded lower droplet diameters, higher droplet density, and better uniformity and penetration than manual spraying. Moreover, conducting UAV spraying twice showed no statistical differences in droplet area percentage compared to manual spraying, and the effect of UAV spraying even once was similar to that of manual spraying on the abaxial sides of the leaves in this respect. Our research indicates that UAV spraying was not very suitable for VSP trellis systems, but it could be a good alternative for Y-shaped trellis systems since it is safe and cost-effective by reducing labor, time, and the dosages of the solutions applied.

## 1. Introduction

Since old times, crops have always been threatened by plant diseases, insect pests and weeds. Based on the Food and Agriculture Organization (FAO) data, the annual loss due to the above-mentioned threats accounts for nearly 40% of the total crop output in the world [1], of which 10% is lost due to diseases, 14% is lost due to pests and 11% is lost due to weed damage. Moreover, as the global population ages, the decrease in the labor force and the aggravation of environmental pollution caused by the abuse of phytosanitary products have gradually worsened the situation [2]. In response to these problems, especially the abuse of phytosanitary products, some countries have put forward the concept of precision agriculture to guide agricultural field practices, aiming to reduce the use of fungicides and pesticides while improving the effectiveness of spraying [3]. Pesticide control is currently dominated by liquid forms, which is considered to pollute the environment but is still the least costly and most efficient way to control crop pests and diseases [4,5]. Thus, more and more attention has been paid to changing the spraying method [5,6]. Traditional spraying methods include knapsack electric sprayer and spray gun, however, they tend to bring a series of problems such as excessive time and labor requirements, as well as waste of fungicides and pesticides [7]. In this context, a high-efficiency and low-labor-intensive spraying method is urgently needed to make agriculture more sustainable.

As advances of photoelectric technology is increasing, aerial spraying technology is gradually applied in agriculture. This technology is composed of flight systems, ground control system and communication system [3,8,9]. The aerial spraying methods get rid of the restrictions of terrain, improving the application efficiency, which leads to reduction in the applied spraying amount while not affecting and soil conditions compared to ground spraying methods [3]. The common aerial spraying methods include helicopter spraying, manned fixed-wing spraying, and rotary-wing unmanned aerial vehicle (UAV) spraying [6,9]. Unlike helicopter spraying, the rotorcraft UAV spraying is suitable for small areas at low altitude. The rotorcraft UAV has the advantage of low turning radius and efficient spraying for specific areas (i.e., complex terrain and small plots) [5,10], without requiring a specific take-off point. In addition, with this spraying method, the operation time is greatly reduced [5,11]. In comparison to manned craft, which exposes pilots to dangerous chemicals, the ground operator can keep a safe distance from the UAV during the operation, which can effectively reduce or even avoid the damage that phytosanitary products could affect the operator [12,13,14]. Therefore, in small farmland and variable plots, rotorcraft drones could be advantageous. Since it is a recent emerging agricultural practice, there are still some problems to be solved, such as the low penetration due to the wind, the low droplet deposition rate or the droplet drift [10,15,16]. In the UAV spraying methods, droplets have several places to go: (1) on the target area, which is called droplet deposition; (2) off target, which is called droplet drift; (3) evaporation; the droplets evaporate and are released into the atmosphere. The purpose of UAV spraying research is to increase droplet deposition and to reduce droplet drift, improving spraying effectiveness.

At today, the studies about UAV spraying in field trials are mainly focused on: (1) UAV operating parameters, such as altitude, speed, spraying amount and flight path [16,17,18]; (2) comparison of different nozzle types on the UAV [15,19]; (3) effectiveness according to canopy characteristics, such as different trellis systems or different parts of canopy; (4) the real pest control effects compared to manual methods. To our knowledge, the previous studies on aerial spraying are mainly focused on the UAV spraying methods on grain, fiber and vegetable farmland, such as rice [20], wheat [21], cotton [22], pepper [23], and other crops. To our knowledge, there are only scarce studies that broaden the application of UAV spraying in vineyards. Sarri et al. [3] compared the spraying performance of commercial drones equipped with different nozzles in high-slope terrace vineyards. The results showed that the working capacity of commercial drones was twice that of spray guns and 1.6 times that of knapsack sprayers. However, the low droplet deposition rate of commercial UAVs indicates that UAVs are still in the early stages of spraying applications, and further research is needed to improve their spraying effects. Giles and Billing [24,25] compared the spraying effects of UAV, manned aerial spraying and ground spraying in vineyards and there was no statistical difference in the droplet deposition rate between UAV and manned aerial spraying. In addition, UAV proved to be more efficient than ground spraying, and the deposition amount obtained by UAV at 47 L/ha flow rate was similar to that obtained by ground spraying (935 L/ha) on the canopy. Sassu et al. [26] studied the impact of different UAV flight heights, flight speeds, and positions (over the canopy and the inter-row) on spraying efficiency in vineyards. The results showed that the best spraying effect was achieved when the UAV flew at a height of 2 m above the ground, with a cruising speed of 1.5 m/s, positioned in the inter-row. Nevertheless, these studies on aerial spraying are mostly focused on the adaxial side of leaves, and to our knowledge, there are few studies that have been carried out evaluating its effects on the abaxial side of leaf. The plant leaves absorb liquid mainly through stomata and hydrophilic pores, and the number of stomata on the abaxial side is higher than that on the adaxial side. Moreover, the thickness of the cuticles on the abaxial side is thinner while the critical surface tension is larger, which makes it easier to attach and absorb droplets [27,28].

Around the world, there are high-slope terrace vineyards, where ground spraying equipment is difficult to go through in most cases [29]. Therefore, the application of UAV spraying could be a good alternative in such vineyards. However, the use of UAV spraying methods in these vineyards is still low, and most viticulturists prefer to use manual spraying for pest control and foliar fertilization. The reasons for this include the high investment cost of drones and the need for certain control skills, yet more importantly, there is little scientific research that evaluates the effectiveness of UAV spraying in vineyards compared to traditional spraying methods, thus people are uncertain if buying a drone for spraying is really useful.

Therefore, the spraying effects of a four-rotor UAV and a knapsack electric sprayer were compared in two vines trellis systems (vertical shoot positioning vs. Y-shaped) in high-slope terrace vineyards. By investigating the influence of different factors such as leaf surface position (adaxial surface or abaxial surface) and canopy height on the spraying effects, the effectiveness and feasibility of UAV spraying in high-slope terrace vineyards were comprehensively evaluated.

## 2. Materials and Methods

### 2.1. Experimental Fields

**Figure 1:**
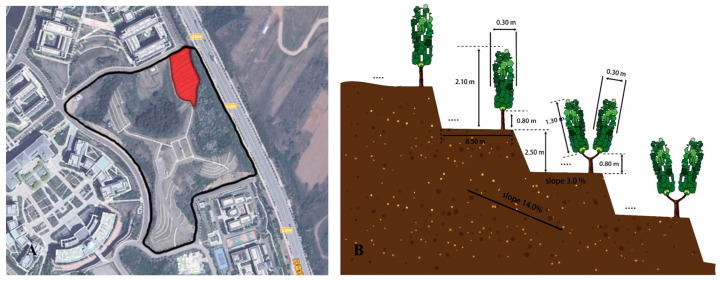
Satellite image of the grapevine vineyard at Shenyang Pharmaceutical University (**A**). In Subfigure A, the black frame indicates the area where grapevines are cultivated by the university, while the red frame marks the experimental grapevine area used in this study. Schematic diagram of the Vidal Blanc plot (**B**).

This study was carried out in the vineyard of Shenyang Pharmaceutical University (41.4594° N, 123.6989° E, Benxi, China). This area has a temperate continental monsoon climate, with warm and humid conditions in the summer. In 2018, Vidal Blanc (Ugni Blanc × Rayon d’Or) vines were planted in a north–south orientation on terraces, which were constructed on a slope of 14%. The height difference between the adjacent terraces was 2.5 m, and the width of each terrace was 8.5 m. The spacing between rows and vines was 2.5 m × 1.0 m, respectively, leaving 3 vine rows spread out over each terrace. As shown in Figure 1, after shoot topping, the width of the canopy was 0.3 m, and the bottom of the canopy was 0.8 m from the ground. Vertical shoot positioning (VSP) and a Y-shaped trellis system were employed on different terraces. The height of the canopy was 1.3 m for the vines arranged on the VSP trellis system, so the top layer of the canopy was 2.1 m from the ground. The canopy was tilted at an angle of 70° to the ground for the vines grown on the Y-shaped trellis system, and the apex of the canopy was about 2.0 m from the ground.

### 2.2. Parameters of Four-Rotor UAV and Knapsack Electric Sprayer (KES)

A T10 Plant Protection UAV (3WWDZ-10A, Shenzhen DJI Innovation Technology Co., Ltd., Shenzhen, China) was used in the research trial. It is an electrically powered four-rotor drone with overall dimensions of 1958 mm × 1833 mm × 553 mm. Its working voltage is 58.8 V, and it weighs 26.8 kg when the solution pot is filled with 10 L of liquid. A GNSS (global navigation satellite system) assisted by RTK (real-time kinematic) allowed us to perform centimeter-level operation planning, and the accuracy of horizontal and vertical operation was ± 10 cm. The drone was equipped with omnidirectional obstacle avoidance radar, with the radar ball showing the distance between the aircraft and an obstacle. A ground remote control system was used to operate the UAV, and the operation situation was updated in real time. The UAV was equipped with four nozzles (type SX11001VS) with a spray pressure of 0.2–0.4 MPa and a spray width of 3 m. The maximum flow rate of the UAV was set at 1.8 L/min. Additionally, a knapsack electronic sprayer (KES) (3WD-20, Shenyang Runfeng Agricultural Machinery Co., LTD., Shenyang, China) with dimensions of 370 mm ×180 mm × 515 mm was used for traditional manual spraying. This machine can carry 20 L of solution and is powered by a 12 V battery. The KES was equipped with a cone nozzle, and the length of the spray rod was 800 mm. The spray pressure was calibrated between 0.40 and 0.45 MPa, leading to a spray volume of 1.2 L/min. Once this device is fully charged, it can operate continuously for eight hours. Table 1 shows the technical parameters of the UAV and KES used in the experiment.

### 2.3. Route Planning for UAV Spraying

**Figure 2:**
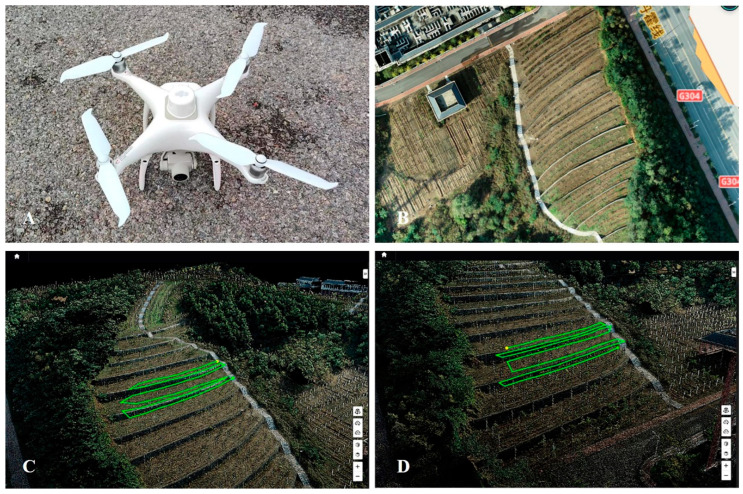
The planning of UAV flight routes. DJI Phantom 4 RTK (**A**); The two-dimensional reconstruction map of the Vidal Blanc plot (**B**); The three-dimensional flight path map of the vines conducted on vertical shoot position (VSP) trellis system (**C**); The three-dimensional flight path map of the vines conducted on Y-shaped trellis system (**D**).

Unlike field crops, the distribution of the vine canopy in a vineyard is discontinuous. Therefore, the spraying route followed by a UAV should be completely in line with the rows of vines to avoid wasting solution. Thus, in this study, the route was drawn manually. Two-dimensional orthophotography was employed using Phantom 4 RTK (Shenzhen DJI Innovation Technology Co., Ltd., Shenzhen, China), and then a digital surface model (DSM) of the experiment patch was obtained using DJI Terra (version 3.4.0, Shenzhen DJI Innovation Technology Co., Ltd., Shenzhen, China). Then, based on DSM, the Phantom 4 RTK was flown once again in terrain awareness mode in the vineyard at a relative height of 40 m. Subsequently, two- and three-dimensional models for route planning were obtained through the processing of the DJI Terra data. At this stage, the “Agricultural Application” function of DJI Terra was used to plan the route. The spraying type was set to “Continuous Spraying”, and the route-planning type was set “Manual”. Routes were created very carefully using the two-dimensional map and validated using the three-dimensional map, and spraying routes were mapped out precisely by using reference points and correcting the ground object recognition results (Figure 2). The height and smoothness of the routes were set to 1.5 m (the lowest height permitted) and 0.5 m, respectively. Finally, the routes were uploaded to the DJI Agricultural Management Platform. The controller of the T10 drone could download the routes before executing the tasks.

### 2.4. Placement of Water Sensitive Paper (WSP) and Spraying Process

**Figure 3:**
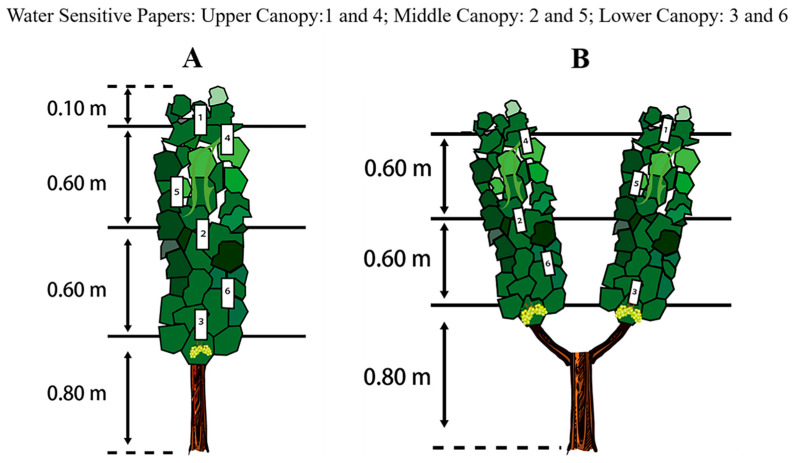
Distribution of water sensitive paper (WSP) within the vine canopy on vertical shoot positioning (VSP) trellis system (**A**) and Y-shaped trellis system (**B**). In upper, middle and lower canopies, two WSPs were pasted to the adaxial side of a leaf and the abaxial side of another leaf, respectively.

**Figure 4:**
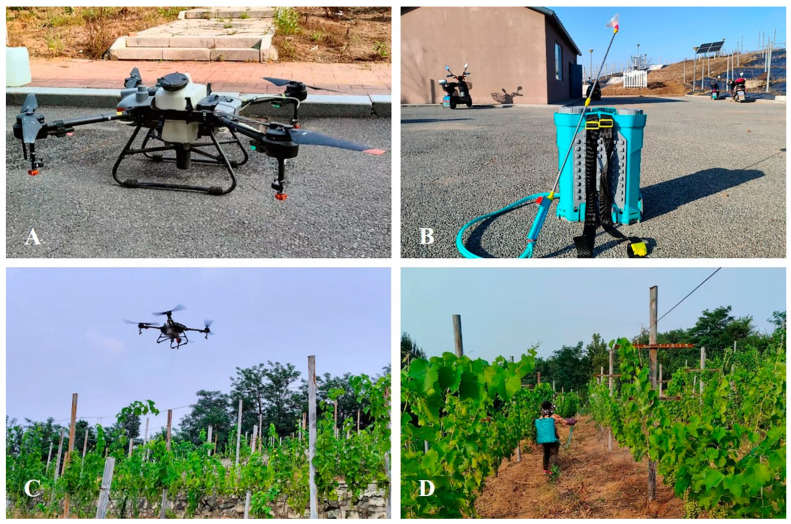
Pictures taken during the spraying process. The four-rotor unmanned aerial vehicle (UAV) DJI T10 (**A**); The knapsack electric sprayer (KES) sprayer (**B**). The spraying process of UAV and manual treatments, respectively (**C**,**D**).

Spraying performance was assessed separately for the vines grown using the vertical shoot position (VSP) and Y-shaped trellis systems. For both groups, three spraying treatments were applied: (i) UAV spraying once (USO); (ii) UAV spraying twice (UST); and (iii) manual spraying (MS). For each treatment, five vines of similar vigor but from different rows were selected as sample vines. In order to strictly control the consistency of spraying, no vines were sampled from the border rows within each treatment zone. Water-sensitive paper (WSP) was used to evaluate and compare the effects of the different spraying treatments. In this study, WSP measuring 35 mm × 55 mm (Chongqing Liuliushanxia Plant Protection Technology Co., LTD., Chongqing, China) was used, and on each vine, six pieces of WSP were pasted with double-sided tape onto six leaves within the canopy. The treatments were evaluated at three canopy heights (upper, middle, and lower canopy), and we chose two leaves on which to apply the WSP (Figure 3). In addition, the effectiveness on leaf surface position (adaxial and abaxial surfaces) was also evaluated. Thus, one of the pasted leaves had WSP on its adaxial side, and the other had it on its abaxial side. UAV spraying proceeded at a relative height of 1.5 m from the top layer of the canopy, with a forward speed of 1 m/s and a flow rate of 1.8 L/min (Figure 4). For the MS treatment, the nozzle of the knapsack electric sprayer (KES) was placed 50 cm away from the leaf canopy, and the operator’s movement speed was 0.5 m/s. Additionally, to ensure operational standardization, the operators were rigorously trained, and preliminary experiments were conducted to determine the optimal spraying method. The operators followed the same predefined path and maintained a consistent walking speed. For uniform application, each operator moved the nozzle up and down to evenly spray the leaves and walked back and forth to ensure that both sides of the canopy were sprayed. Tap water was used in all the treatments, and the test was performed from 10:00 to 16:00 h to reduce the influence of moisture and temperature. At the end of each treatment, we waited at least 5 min before removing the pieces of WSP to ensure that they were completely dry. Then, once removed, the pieces of WSP were stored in a self-sealing bag and quickly transferred to our laboratory for further scanning and image analyses. Meteorological data were recorded in detail during all the spraying treatments (Table 2). Specifically, a hand-held anemometer (ZTW1801B, China Network Technology Co., LTD., Hangzhou, China) and an industrial hygrograph (THM-01, Delxi Electric Co., LTD., Wenzhou, China) were used to measure wind speed (m/s), wind direction, temperature (°C) and relative humidity (%). During the test period, the average temperature ranged from 32.8 to 35.4 °C, the average relative humidity ranged from 42.8 to 55.9%, and the wind speed ranged from 0.9 to 1.8 m/s, with east and southeast being the predominant orientation.

### 2.5. Image Processing and Analysis

**Figure 5:**
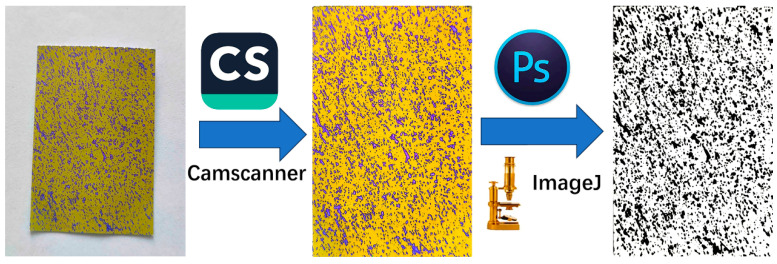
The binary image obtained using the ImageJ software that allowed to obtain the droplet area percentage, the droplet density and the droplet diameter.

In a dark room, all the pieces of WSP were photographed with a fixed phone on flash in order to obtain photos of similar quality for subsequent batch analyses. Subsequently, all the WSP images were imported into CamScanner software (version 6.87.0.2504250000, Shanghai Hehe Information Co., LTD., Shanghai, China) to automatically cut the edges of the pieces of WSP. For each image, the blue parts of the WSP were selected and replaced with black by using Photoshop software (version 22.1.1, Adobe Systems Incorporated, San Jose, CA, USA), while the rest of the picture was rendered white. Consequently, binary images of the WSP were acquired and saved as JPEG files. The binary images were analyzed with ImageJ software (version 1.53e, National Institutes of Health, Bethesda, MD, USA) to obtain the droplet area percentage, the droplet density, and the droplet diameter [30]. The droplet area percentage is the proportion of the droplet area to the total area of the water-sensitive paper. Droplet density is the number of droplets per unit area on the water-sensitive paper. The droplet average diameter is the sum of the diameters of all droplets on the water-sensitive paper divided by the number of droplets. The process of WSP image processing is shown in Figure 5. Moreover, the coefficient of variation (CV) was used to evaluate droplet uniformity and penetration by each treatment. The CV of droplet density at different sampling points at each height was used to calculate droplet deposition uniformity [16,17]. The CV of the droplet density among the upper, middle, and lower layers indicates the penetration degree. In general, a small CV means good uniformity and penetration [31]. The formula for calculating CV is as follows:

Equation (1). The coefficient of variation calculation of droplet density.

(1) $$CV=\frac{SD}{\bar{X}}\times 100\%$$

$$\bar{X}=\sum_{i=1}^{n}\frac{X_{i}}{n}\;\;\;SD=\sqrt{\sum_{i=1}^{n}\frac{{\left(X_{i}-\bar{X}\right)}^{2}}{n-1}}$$

Here, $X_{i}$ represents the droplet density of each piece of WSP sampled; $\bar{X}$ represents the mean of the droplet densities of all the pieces of WSP sampled; n represents the number of pieces of WSP sampled; and SD represents the standard deviation of all the samples.

### 2.6. Statistical Analysis

All data were statistically analyzed using IBM SPSS26 (IBM Corp., Armonk, NY, USA). Three-factor analysis of variance (TANOVA) was used to study the effects of spraying method, canopy height, and leaf surface position on droplet area percentage, droplet density, and droplet size. The Duncan test was used to conduct multiple post hoc comparisons, and simple effect analysis was used to analyze the second-order interaction effects. Finally, the R language (R Core Team, New Zealand) and GraphPad Prism software (version 8.0.2 (263), Graphpad Corp., San Diego, CA, USA) were used to create graphs.

## 3. Results and Discussion

### 3.1. Effects of Treatments on Vertical Shoot Positioning (VSP) Trellis System Vines

We performed a three-factor analysis of variance (ANOVA) to evaluate the effects of the spraying method (UAV and KES), leaf surface position (the states of the adaxial and abaxial sides), and canopy height (upper, middle, and lower canopy) on droplet average diameter, droplet area percentage, and droplet density for vines grown using a vertical shoot positioning (VSP) trellis system, as shown in Table 3. In Table 3, degrees of freedom (DF) refer to the number of independent values that can vary in a statistical calculation; the F-statistic (F) is used to compare the variance between groups to the variance within groups, helping reveal whether the means are significantly different; and the *p*-value (*p*) indicates the probability of observing a given statistic (or something more extreme) assuming that the null hypothesis is true.

**Figure 6:**
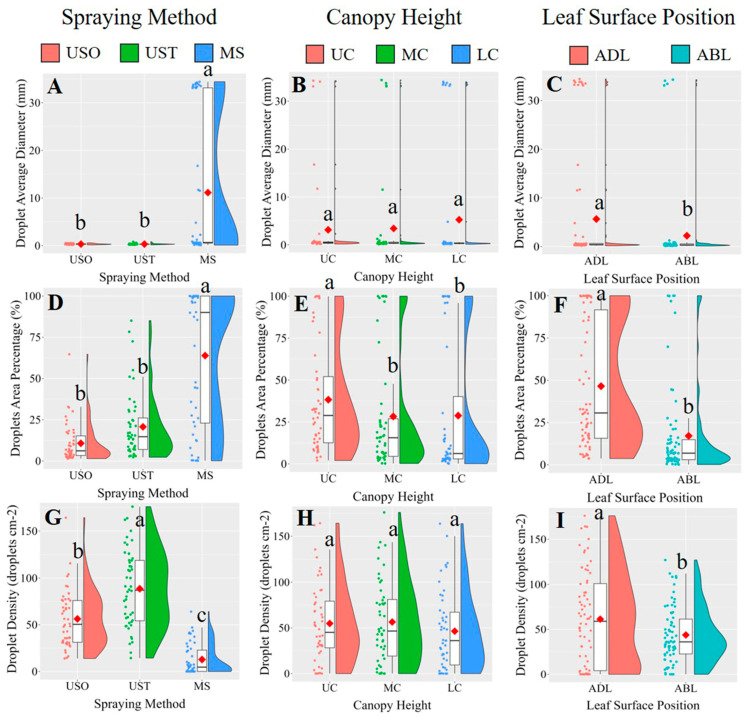
A multiple comparison of three factors (spraying method, canopy height and leaf surface position) was conducted for droplet average diameter (mm), droplets area percentage (%) and droplet density (droplets cm^−2^) in the VSP trellis system. Scatter plots, box plots, and half-violin plots were utilized to visualize the data more clearly, with red-filled ◇ indicating the mean of each dataset. Data were analyzed with a Duncan test (*α* = 0.05), different letters (a, b) above box plots represent significant differences. VSP: Vertical shoot positioning. USO: UAV spraying once. UST: UAV spraying twice. MS: Manual spraying. UC: Upper canopy. MC: Middle canopy. LC: Lower canopy. ADL: Adaxial surface of leaf. ABL: Abaxial surface of leaf. Subfigures (**A**,**D**,**G**) illustrate the comparisons of droplet average diameter (mm), droplet area percentage (%), and droplet density (droplets cm^−2^) under different spraying methods. Subfigures (**B**,**E**,**H**) show the comparisons of droplet average diameter (mm), droplet area percentage (%), and droplet density (droplets cm^−2^) at different canopy heights. Subfigures (**C**,**F**,**I**) present the comparisons of droplet average diameter (mm), droplet area percentage (%), and droplet density (droplets cm^−2^) between the adaxial and abaxial leaf surfaces.

**Figure 7:**
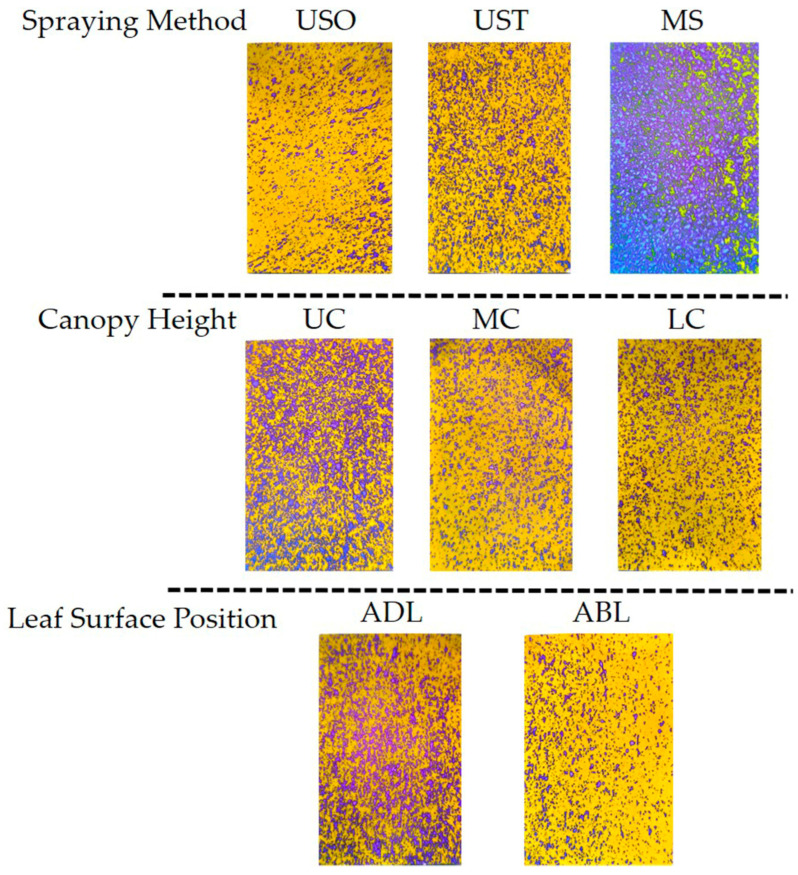
Schematic diagram of water-sensitive paper under different treatments in the VSP trellis system. USO: UAV spraying once. UST: UAV spraying twice. MS: Manual spraying. UC: Upper canopy. MC: Middle canopy. LC: Lower canopy. ADL: Adaxial surface of leaf. ABL: Abaxial surface of leaf.

The results showed that spraying method and leaf surface position significantly affected droplet average diameter, droplet area percentage, and droplet density in VSP trellis system vines. Canopy height only significantly affected droplet area percentage. The results of the post-hoc multiple comparison test (Figure 6) showed that UAV spraying resulted in significantly smaller droplets and grater droplet densities. The difference in droplet size might be due to the fact that the leaves were sprayed with the KES nozzles for a greater duration than under UAV spraying because of the limited speed of the equipment. Accordingly, the larger droplets slide off the leaves more easily, contributing to the wastage of the spray liquid [9]. MS yielded a higher average droplet area percentage than the UAV method (63.9% versus 10.74% for USO and 20.72% for UST), indicating that MS led to a better application area on the leaves than the UAV spraying method. This result is consistent with the findings of Xiao et al. [23], who reported that a manual spraying method yielded a higher droplet coverage rate than UAV methods. The average droplet area was 46.49% for the adaxial side of the leaf (ADL) and 17.08% for the abaxial side of the leaf (ABL), meaning the ADL received much more liquid than the ABL. On the surface of a plant, solutions are mainly absorbed by the cuticle, cuticular stacks, stomata, trichomes, and lenticels, so it is possible that a considerable amount of the solution used in vineyards does not reach the vine canopy. Accordingly, in this study, the upper canopy (UC) received significantly more spray liquid than the lower parts, accounting for 35.5% more than the middle canopy (MC) and 33.0% more than the lower canopy (LC). These results indicate that droplets are more likely to deposit on the upper layers of the vines as opposed to the lower ones. These findings are similar to those obtained by Qin et al. [6], who showed that droplets tend to be deposited to a greater extent on the upper canopy in UAV and ground-spraying methods. Samples of the original images in the VSP trellis system are shown in Figure 7.

Table 4 shows the effects of the interaction of the factors influencing the spraying method, leaf surface position, and canopy height on droplet average diameter, droplet area percentage, and droplet density in regard to the VSP trellis system vines. Spraying method and leaf surface position significantly affected droplet average diameter, droplet area percentage, and droplet density, while the interaction between spraying method and canopy height only affected droplet area percentage. The rest of the interactions did not significantly affect the parameters measured in this field trial.

**Figure 8:**
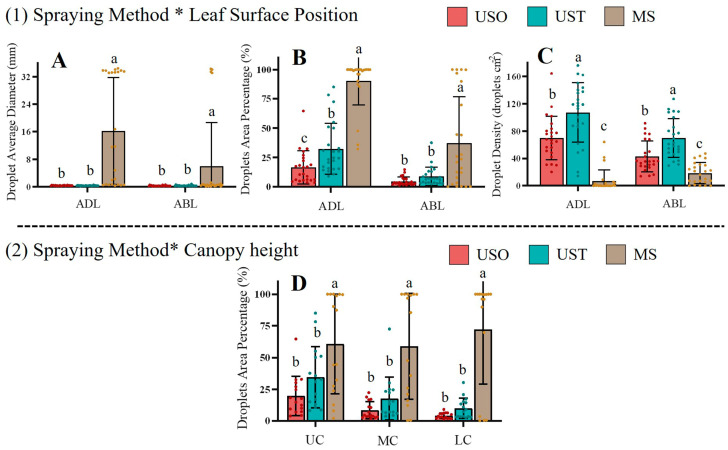
The simple effects analysis between (1) Spraying method * Leaf surface position and (2) Spraying method * Canopy height in the VSP trellis system vines. Data were analyzed with a Duncan test (*α* = 0.05), different letters (a, b, c) represent significant differences. VSP: Vertical shoot positioning. USO: UAV spraying once. UST: UAV spraying twice. MS: Manual spraying. UC: Upper canopy. MC: Middle canopy. LC: Lower canopy. ADL: Adaxial surface of leaf. ABL: Abaxial surface of leaf. Subfigures (**A**–**C**) illustrate the effects of different spraying methods on droplet average diameter (mm), droplet area percentage (%), and droplet density (droplets cm^−2^) with respect to the leaf surface position. Subfigure (**D**) show the effects of different spraying methods on droplet area percentage (%) with respect to the canopy height.

Figure 8 shows the effects of the spraying methods on droplet average diameter, droplet area percentage, and droplet density with respect to the ADL and ABL parameters of the VSP trellis system vines. For ADL and ABL, MS yielded the maximum droplet diameters: 16.25 mm for the ADL and 6.04 mm for the ABL. In contrast, the droplet diameter for USO and UST ranged from 0.30 to 0.37 mm, indicating that UAV spraying deposited smaller droplets on the vine leaves than MS, which might favor their absorption of the droplets. Accordingly, although the diameters of the droplets (blue stains) on both sides of the leaves subjected to MS treatment were much larger than the diameters of the droplets deposited via UAV, this gap was especially pronounced on the adaxial leaf surface. Nevertheless, MS treatment exhibited high variability in the measured parameters. Thus, based on leaf morphology, the large sizes of the droplets (blue stains) deposited via MS could hinder the absorption of solutions, mostly for the adaxial leaf surface. Nevertheless, the MS treatment exhibited advantages related to its effects on average droplet area, reaching percentages of 90.33% for the ADL and 37.47% for the ABL. There were significant differences between the USO and UST treatments with respect to the adaxial side of the leaf; this trend was not significant on the abaxial side. Regarding the VSP trellis system vines, it was more difficult to deposit droplets on the abaxial sides of the leaves using the UAV spaying method in comparison to the MS treatments. The vertical downforce wind field created by the UAV sprayers prevented the adequate deposition of droplets on the abaxial sides of the leaves in the VSP trellis system vines because of the vertical structure of this type of system. However, in the MS treatment, it was also difficult to deposit the solution on the abaxial sides of the vine leaves. The UAV and MS treatments showed significant differences in terms of droplet density on both sides of the vine leaves. The larger droplet density brought about by UAV spraying could indicate more points of action [32,33]. MS treatment deposited a significantly larger area of droplets than UAV spraying at different canopy heights for the VSP trellis system vines (Figure 8D).

The results of spraying uniformity and penetrability are displayed in Table 5. Given that the values of these two indicators are small in USO and UST spraying applications, it is possible to infer that UAV spraying gave rise to a much better uniformity at each canopy height level, and also to a better penetrability than the manual spraying method [17,31].

### 3.2. Effects of Treatments on Y-Shaped Trellis System Vines

Table 6 shows the effects of the spraying method (UAV and KES), leaf surface position (adaxial and abaxial sides), and canopy height (upper, middle, and lower canopy) on droplet average diameter, droplet area percentage, and droplet density for Y-shaped trellis system vines. The results show that the spraying method significantly affected droplet average diameter, droplet area percentage, and droplet density for the Y-shaped trellis system vine leaves, whereas leaf surface position significantly affected droplet average diameter and droplet density. Canopy height only significantly affected droplet area percentage for the VSP trellis system vines.

**Figure 9:**
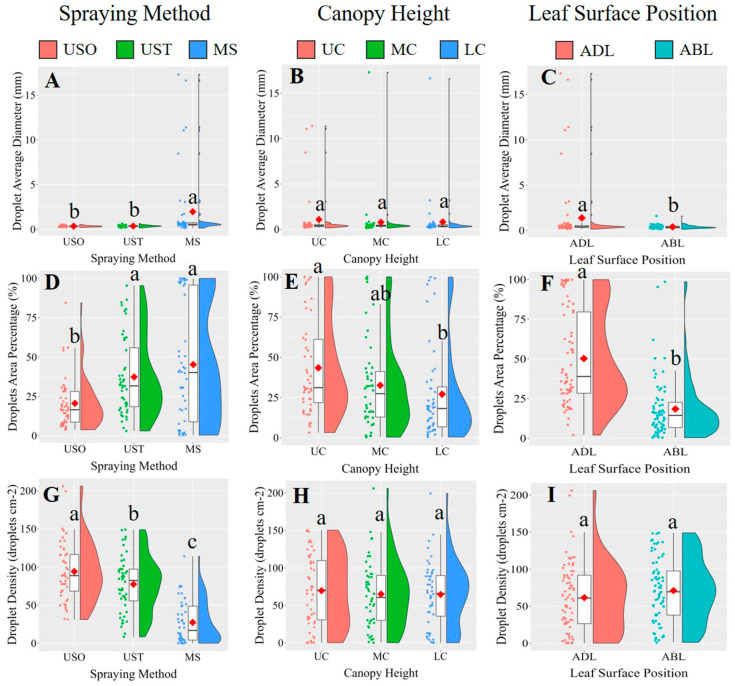
A multiple comparison of three factors (spraying method, canopy height and leaf surface position) was conducted for droplet average diameter (mm), droplets area percentage (%) and droplet density (droplets cm^−2^) in the Y-shaped trellis system. Data visualization was facilitated through scatter plots, box plots, and half-violin plots, with the mean of each dataset highlighted using red-filled ◇. Data were analyzed with a Duncan test (*α* = 0.05), different letters (a, b, c) above box plots represent significant differences. USO: UAV spraying once. UST: UAV spraying twice. MS: Manual spraying. UC: Upper canopy. MC: Middle canopy. LC: Lower canopy. ADL: Adaxial surface of leaf. ABL: Abaxial surface of leaf. Subfigures (**A**,**D**,**G**) illustrate the comparisons of droplet average diameter (mm), droplet area percentage (%), and droplet density (droplets cm^−2^) under different spraying methods. Subfigures (**B**,**E**,**H**) show the comparisons of droplet average diameter (mm), droplet area percentage (%), and droplet density (droplets cm^−2^) at different canopy heights. Subfigures (**C**,**F**,**I**) present the comparisons of droplet average diameter (mm), droplet area percentage (%), and droplet density (droplets cm^−2^) between the adaxial and abaxial leaf surfaces.

**Figure 10:**
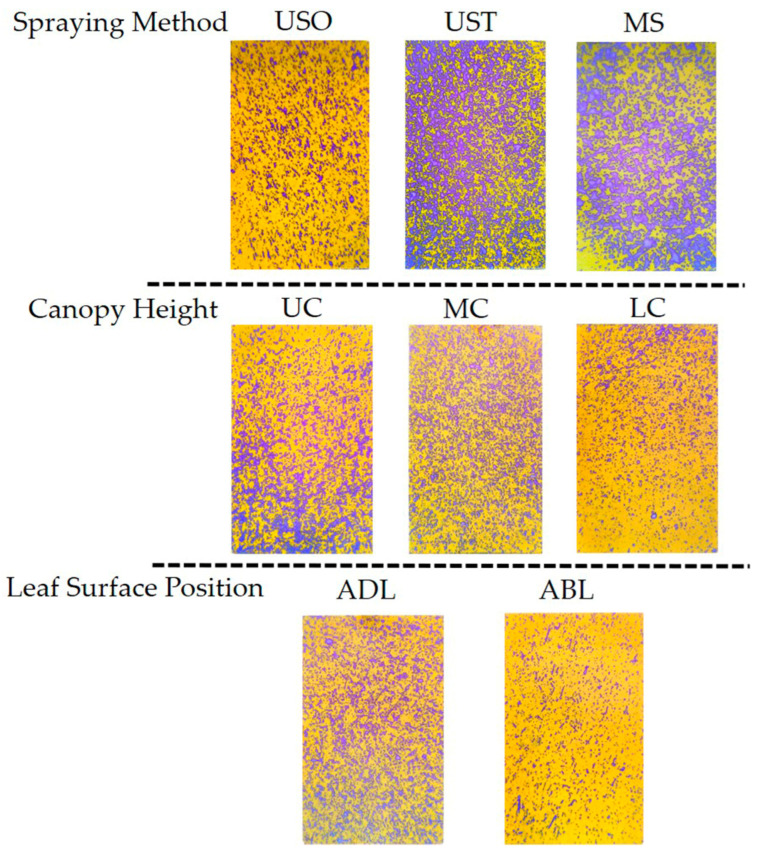
Diagram illustrating the effects of different treatments on water-sensitive paper in Y-shaped trellis system. USO: UAV spraying once. UST: UAV spraying twice. MS: Manual spraying. UC: Upper canopy. MC: Middle canopy. LC: Lower canopy. ADL: Adaxial surface of leaf. ABL: Abaxial surface of leaf.

Figure 9 shows that the MS treatment yielded a significantly higher droplet diameter than the UAV spraying method. The leaf surface position factor also affected this parameter since the average droplet diameter on the adaxial side of the leaf (ADL) was higher than that on the abaxial side (ABL). The trends of these results are similar to those found for the VSP trellis system vines. Similar to the VSP trellis system vines, the USO treatment covered significantly less area than the MS and UST treatments (Figure 9D). This finding is important since the droplet coverage percentage is the most important indicator for evaluating spraying performance. The droplet area percentages of the upper and lower Y-shaped trellis system canopy were statistically different, reaching 43.29% and 27.10%, respectively. Spraying method had a statistically significant effect on droplet density but not leaf surface position or canopy height (Figure 9G–I). The droplet densities for the USO, UST, and MS treatments were 94.32 droplets cm^−2^, 77.54 droplets cm^−2^, and 27.36 droplets cm^−2^, respectively, contrary to the trend observed for the VSP trellis system vines, for which UST led to a greater droplet density than USO. This result could be due to the better penetration of droplets in the canopy of the Y-shaped trellis system in comparison to that of the VSP trellis system, since, in the UST treatment, the droplets of the second spraying combined with those from the first spraying converged into larger droplets, reducing the density of the droplets. Samples of the original images in the Y-shaped trellis system are shown in Figure 10.

**Figure 11:**
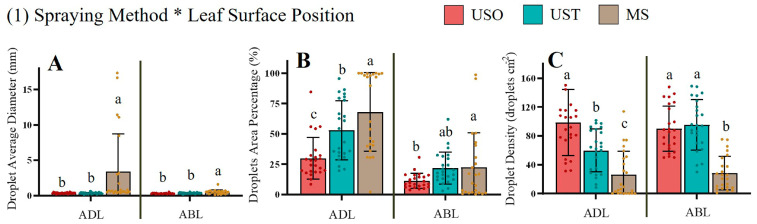
The simple effects analysis between Spraying method * Leaf surface position in the Y-shaped trellis system vines. Data were analyzed with a Duncan test (*α* = 0.05), different letters (a, b, c) represent significant differences. USO: UAV spraying once. UST: UAV spraying twice. MS: Manual spraying. ADL: Adaxial surface of leaf. ABL: Abaxial surface of leaf. Subfigures (**A**–**C**) illustrate the effects of different spraying methods on droplet average diameter (mm), droplet area percentage (%), and droplet density (droplets cm^−2^) with respect to the leaf surface position.

Table 7 shows that only the interaction between the spraying method and leaf surface position factors affected the parameters measured. Figure 11 shows that although the MS treatment led to the highest average droplet diameter on both leaf sides (3.43 mm on the ADL and 0.52 mm on the ABL), the difference between the MS and UAV spraying methods was much greater in the case of the ADL, which means that during manual spraying, a large number of droplets could gather on leaf adaxial side and be lost easily, potentially resulting in the wastage of the solution. There were significant differences between the treatments in terms of the droplet area percentages on the adaxial and abaxial sides of the leaves (Figure 11B). Manual spraying resulted in the highest droplet area on the adaxial side, whereas UST resulted in a lower covered area than the MS treatment on the adaxial side. Regarding UAV spraying, the Y-shaped trellis system vines exhibited a higher droplet area than that of the VSP trellis system vines, whereas for MS, the percentage was lower for the Y-shaped trellis system vines. Thus, it is possible that the UAV spraying performance was closer to that of the MS treatment for the Y-shaped trellis system on the adaxial side in comparison to the VSP trellis system. The effects of the UST and MS treatments were statistically similar in terms of the droplet percentage area on the abaxial side of the leaves. Since stomata in vines are mostly located on the abaxial side of the leaf, the absorption efficiency on this side under the UST treatment could be similar to that of the MS treatment when applied to the Y-shaped trellis system. Manual spraying yielded a significantly lower droplet density than UAV spraying. Between UAV treatments, USO yielded a significantly higher droplet density than UST on the adaxial side but not the abaxial one, possibly because of the partial overlap of the droplets that occurred in the UST treatment, potentially leading to a reduced droplet density on the leaves’ adaxial side.

Table 8 shows that the droplet uniformity on the abaxial side of the leaves was better than that on the adaxial one for the Y-shaped trellis system vines in most of the applications. This result could be due to the fact that grapevine leaves of the Vidal Blanc variety exhibit strong phototropism, and the surface of the canopy could have greater exposure to the spraying of droplets since the canopy is mostly exposed, on the adaxial side of the leaves, to the environment. On the other hand, in the inner canopy, both the adaxial and abaxial sides of the leaves are exposed. Thus, the variability among the abaxial sides of the leaves is likely to be higher. In addition, UAV spraying yielded better results than MS treatment in terms of uniformity and penetration, as previously shown for VSP trellis system vines. Meng et al. [7] reported similar results for Y-shaped trellis system peach trees based on UAV operational parameters. Their results indicated that Y-shaped trellis system peach trees exhibited better uniformity in inner-layer positions when UAV sprayers were used.

### 3.3. Comparative Analysis of Efficiency Between Manual Spraying and Unmanned Aerial Vehicle (UAV) Sprayings

The above values represent only the average values measured or calculated under the experimental conditions of this study. If the growing pattern (vine row length, the space between rows or terraces, etc.) changed, the values would vary slightly.

The decision of whether to use UAVs to apply spraying solutions in vineyards should incorporate an assessment of the effectiveness of spraying, including the operational efficiency of the equipment employed. Table 9 shows the average values of the different working efficiency determinations measured and calculated in this field trial. The effective spraying time for UAV applications refers to the time taken by the drone to actually spray all the vine rows. Accordingly, the additional time for UAV spraying includes the total amount of time required for take-off, the flight path to target area, battery replacement, spray tank filling, and landing. The additional time for MS application considers the time taken to fill the tank and reach the target area. The total operation time is equal to the sum of the effective time and the additional time. Working area per hour is the reciprocal of the total operation time per hectare, and the spray volume per hectare is the product of the flow rate and the effective spraying time per unit time. Based on Table 9, it only takes 1.96 h and 3.92 h for the USO and UST to be applied to 1 hectare, respectively, with the working efficiency being 6 and 3 times that of MS, respectively. On the other hand, the usage of solution for MS is 504.72 L/ha, about 4 times that of USO and 2 times that of UST. Furthermore, in this study, the UAV nozzles were always open during the spraying process. In future applications, for UAV spraying, the amount of solution could be reduced further by ceasing spraying when the drone moves from one row to another, a process that could be easily realized simply by changing the ground target identification results in DJI Terra. Importantly, the calculation of the efficiency of the MS treatment did not consider the time the workers need to rest; therefore, the actual efficiency gap could be even greater, especially on hot days. Moreover, the safety of workers should not be ignored, especially since manual spraying constantly exposes them to solution droplets that could be toxic. Although UAV spraying is more efficient and labor-saving and safer than manual spraying, the cost of UAV application could be considerable. The economic cost of UAV spraying methods depends mostly on the size of the vineyard. The smaller the vineyard, the smaller the yield, and the higher the average fixed costs. In this case, UAV spraying is not an economical option. However, for a huge vineyard, UAV spraying might be a fairly good alternative to MS.

## 4. Conclusions

In this study, the effects of unmanned aerial vehicle (UAV) spraying and manual spraying via a knapsack electric sprayer (KES) on droplet average diameter, droplet area percentage, and droplet density were compared for vines trellised in vertical shoot positioning (VSP) and grown on a Y-shaped trellis system. Spraying methods, leaf surface position, and canopy were also taken into consideration as variable factors to evaluate their impacts on spraying effectiveness. UAV spraying led to a considerably lower droplet area than manual spraying (MS) for the VSP trellis system vines. Under Y-shaped trellis system, UAV spraying led to smaller droplets with better uniformity and penetrability, consistent with the VSP trellis system results, but at the same time, the coverage rate of droplets was more acceptable than that for the VSP trellis system, especially when the UAV sprayed more than one time; this trend was more obvious, particularly on the abaxial side of the leaves. In general, UAV spraying could be more suitable for Y-shaped trellis systems since its effectiveness was greater than that of manual spraying. Thus, in high-surface-area vineyards that are trellised via a Y-shaped trellis system, UAV spraying would be more economic and effective than manual spraying. This study is the first to investigate the impacts of UAV spraying on the adaxial and abaxial sides of leaves as well as their effects under different trellis systems in high-slope vineyards. Further studies should focus on evaluating the effectiveness and performance of UAV spraying under different trellis systems to aid in the selection of an economic trellis system when establishing a vineyard on difficult terrain that is inaccessible to agricultural machinery.

## Figures and Tables

**Table 1:** Technical parameters of the four-rotor UAV and the knapsack electronic sprayer (KES) used in this research trial.

Technical Parameter	KES	Four-Rotor UAV
Nozzle type	cone nozzle	SX11001VS
Spraying angle	60°	110°
Number of nozzles	1	4
Tank capacity (L)	20	10
Spraying width (m)	1	3
Maximum flow rate (L/min)	1.2	1.8

**Table 2:** Meteorological data recorded during the research trial.

Treatment		Wind Speed (m/s)	Wind Direction	Temperature (°C)	Humidity (%)
Vertical shoot position (VSP) trellis system	USO ^a^	1.8	E	35.4	42.8
	UST ^b^	1.5	E	34.1	43.7
	MS ^c^	0.9	SE	34.6	42.9
Y-shaped trellis system	USO	1.8	E	32.8	51.9
	UST	1.7	SE	33.4	55.9
	MS	1.8	SE	34.9	53.3

^a^ USO: UAV spraying once. ^b^ UST: UAV spraying twice. ^c^ MS: Manual spraying.

**Table 3:** Effects of spraying method (UAV and KES), leaf surface position (adaxial and abaxial side) and canopy height (upper, middle and lower canopy) on droplet average diameter, droplets area percentage and droplet density in vertical shoot positioning (VSP) trellis system vines.

Factors	Test Indicators	DF ^a^	F ^b^	*p* ^c^	Significance ^d^
Spraying method ^e^	Droplet average diameter (mm)	2	26.793	0.000	***
	Droplets area percentage (%)	2	93.214	0.000	***
	Droplet density (droplets cm^−2^)	2	94.511	0.000	***
Leaf surface position ^f^	Droplet average diameter (mm)	1	6.068	0.015	*
	Droplets area percentage (%)	1	75.743	0.000	***
	Droplet density (droplets cm^−2^)	1	15.192	0.000	***
Canopy height ^g^	Droplet average diameter (mm)	2	0.904	0.407	ns
	Droplets area percentage (%)	2	3.718	0.027	*
	Droplet density (droplets cm^−2^)	2	1.910	0.152	ns

^a^ DF means degrees of freedom. ^b^ F means F-statistic. ^c^ *p* means *p*-value. ^d^ statistical significance level: ns, *, *** significant at *p* > 0.05, *p* ≤ 0.05, *p* ≤ 0.001, respectively. ^e^ Spraying method: (1) UAV spraying once; (2) UAV spraying twice; (3) manual spraying. ^f^ Leaf surface position (adaxial surface or abaxial surface): (1) adaxial surface of leaf; (2) abaxial surface of leaf. ^g^ Canopy height: (1) upper canopy; (2) middle canopy; (3) lower canopy.

**Table 4:** The interaction effects of spraying method, leaf surface position and canopy height on droplet average diameter, droplets area percentage and droplet density in VSP trellis system vines.

Source	Test Indicators	DF ^a^	F ^b^	*p* ^c^	Significance ^d^
Spraying method ^e^ * Leaf surface position ^f^	Droplet average diameter (mm)	2	5.938	0.003	**
	Droplets area percentage (%)	2	13.025	0.000	***
	Droplet density (droplets cm^−2^)	2	10.756	0.000	***
Spraying method * Canopy height ^g^	Droplet average diameter (mm)	4	0.979	0.422	ns
	Droplets area percentage (%)	4	3.480	0.010	**
	Droplet density (droplets cm^−2^)	4	2.093	0.086	ns
Leaf surface position * Canopy height	Droplet average diameter (mm)	2	0.148	0.862	ns
	Droplets area percentage (%)	2	1.718	0.184	ns
	Droplet density (droplets cm^−2^)	2	2.318	0.103	ns
Spraying method * Canopy height * Leaf surface position	Droplet average diameter (mm)	4	0.136	0.969	ns
	Droplets area percentage (%)	4	0.302	0.876	ns
	Droplet density (droplets cm^−2^)	4	1.445	0.223	ns

^a^ DF means degrees of freedom. ^b^ F means F-statistic. ^c^ *p* means *p*-value. ^d^ statistical significance level: ns, **, *** significant at *p* > 0.05, *p* ≤ 0.01, *p* ≤ 0.001, respectively. ^e^ Spraying method: (1) UAV spraying once; (2) UAV spraying twice; (3) manual spraying. ^f^ Leaf surface position (adaxial surface or abaxial surface): (1) adaxial surface of leaf; (2) abaxial surface of leaf. ^g^ Canopy height: (1) upper canopy; (2) middle canopy; (3) lower canopy.

**Table 5:** The influence of the spraying methods (UAV spraying once (USO), UAV spraying twice (UST) and manual spraying (MS)) on uniformity and penetrability in vertical shoot positioning (VSP) trellis system vines.

Treatments	Uniformity (%)						Penetrability (%)	
	Upper Canopy		Middle Canopy		Lower Canopy			
	Adaxial Side	Abaxial Side	Adaxial Side	Abaxial Side	Adaxial Side	Abaxial Side	Adaxial Side	Abaxial Side
USO ^a^	49.52	45.09	35.64	56.38	40.72	32.67	45.61	52.32
UST ^b^	59.25	50.49	39.16	37.81	20.64	25.83	40.72	40.75
MS ^c^	219.00	59.56	171.88	94.30	169.12	83.08	228.82	83.57

^a^ USO: UAV spraying once. ^b^ UST: UAV spraying twice. ^c^ MS: Manual spraying.

**Table 6:** Effects of spraying method (UAV and KES), leaf surface position (adaxial and abaxial side) and canopy height (upper, middle and lower canopy) on droplet average diameter, droplets area percentage and droplet density in vines conduced on Y-shaped trellis system.

Source	Test Indicators	DF ^a^	F ^b^	*p* ^c^	Significance ^d^
Spraying method ^e^	Droplet average diameter (mm)	2	8.288	0	***
	Droplets area percentage (%)	2	16.49	0	***
	Droplet density (droplets cm^−2^)	2	51.744	0	***
Leaf surface position ^f^	Droplet average diameter (mm)	1	6.974	0.009	**
	Droplets area percentage (%)	1	78.956	0	***
	Droplet density (droplets cm^−2^)	1	2.925	0.09	ns
Canopy height ^g^	Droplet average diameter (mm)	2	0.265	0.767	ns
	Droplets area percentage (%)	2	7.244	0.001	***
	Droplet density (droplets cm^−2^)	2	0.333	0.717	ns

^a^ DF means degrees of freedom. ^b^ F means F-statistic. ^c^ *p* means *p*-value. ^d^ statistical significance level: ns, **, *** significant at *p* > 0.05, *p* ≤ 0.01, *p* ≤ 0.001, respectively. ^e^ Spraying method: (1) UAV spraying once; (2) UAV spraying twice; (3) manual spraying. ^f^ Leaf surface position (adaxial surface or abaxial surface): (1) adaxial surface of leaf; (2) abaxial surface of leaf. ^g^ Canopy height: (1) upper canopy; (2) middle canopy; (3) lower canopy.

**Table 7:** The interaction effects of spraying method, leaf surface position and canopy height on droplet average diameter, droplets area percentage and droplet density in Y-shaped trellis system vines.

Source	Test Indicators	DF ^a^	F ^b^	*p* ^c^	Significance ^d^
Spraying method ^e^ * Leaf surface position ^f^	Droplet average diameter (mm)	2	6.575	0.002	**
	Droplets area percentage (%)	2	4.7	0.011	*
	Droplet density (droplets cm^−2^)	2	5.633	0.005	**
Spraying method * Canopy height ^g^	Droplet average diameter (mm)	4	0.293	0.882	ns
	Droplets area percentage (%)	4	1.169	0.328	ns
	Droplet density (droplets cm^−2^)	4	0.263	0.901	ns
Leaf surface position * Canopy height	Droplet average diameter (mm)	2	0.182	0.834	ns
	Droplets area percentage (%)	2	0.502	0.606	ns
	Droplet density (droplets cm^−2^)	2	2.802	0.064	ns
Spraying method * Canopy height * Leaf surface position	Droplet average diameter (mm)	4	0.238	0.917	ns
	Droplets area percentage (%)	4	0.193	0.942	ns
	Droplet density (droplets cm^−2^)	4	1.179	0.323	ns

^a^ DF means degrees of freedom. ^b^ F means F-statistic. ^c^ *p* means *p*-value. ^d^ statistical significance level: ns, *, ** significant at *p* > 0.05, *p* ≤ 0.05, *p* ≤ 0.01, respectively. ^e^ Spraying method: (1) UAV spraying once; (2) UAV spraying twice; (3) manual spraying. ^f^ Leaf surface position (adaxial surface or abaxial surface): (1) adaxial surface of leaf; (2) abaxial surface of leaf. ^g^ Canopy height: (1) upper canopy; (2) middle canopy; (3) lower canopy.

**Table 8:** The influence of spraying method (UAV spraying once (USO), UAV spraying twice (UST) and manual spraying (MS)) on uniformity and penetrability in the Y-shaped trellis system vines.

Spraying Method	Uniformity (%)						Penetrability (%)	
	Upper Canopy		Middle Canopy		Lower Canopy			
	Adaxial Side	Abaxial Side	Adaxial Side	Abaxial Side	Adaxial Side	Abaxial Side	Adaxial Side	Abaxial Side
USO ^a^	51.78	25.13	53.39	39.89	38.50	33.69	46.35	34.73
UST ^b^	54.56	36.84	53.42	38.70	38.51	34.13	49.50	36.69
MS ^c^	141.47	75.58	87.52	94.76	126.98	85.99	122.69	82.46

^a^ USO: UAV spraying once. ^b^ UST: UAV spraying twice. ^c^ MS: Manual spraying.

**Table 9:** Comparative analysis of efficiency between manual spraying (MS treatment) and unmanned aerial vehicle spraying methods (USO and UST treatments).

	Manual Spraying (MS)	UAV Spraying Once (USO)	UAV Spraying Twice (UST)
**Efficiency indexes**			
Flow rate (L/min)	1.2	1.8	1.8
Effective spraying time per hectare (h/ha)	7.01	1.14	2.28
Additional time per hectare (h/ha)	1.86	0.82	1.64
Total operation time per hectare (h/ha)	8.87	1.96	3.92
Working area per unit time (ha/h)	0.14	0.88	0.44
Spray volume per hectare (L/ha)	504.72	123.12	246.24

## Data Availability

Data are contained within the article.
